# Difference in left atrial D-dimer level in patients with atrial fibrillation treated with direct oral anticoagulant

**DOI:** 10.1186/s12872-021-02285-y

**Published:** 2021-10-09

**Authors:** Tetsuya Watanabe, Koichi Tachibana, Yukinori Shinoda, Tomoko Minamisaka, Hidetada Fukuoka, Hirooki Inui, Keisuke Ueno, Souki Inoue, Kentaro Mine, Shiro Hoshida

**Affiliations:** 1 Division of Cardiology, Osaka General Medical Center, 3-1-56 Bandai-Higashi, Sumiyoshi-ku, Osaka, 558-8558 Japan; 2 Department of Cardiovascular Medicine, Yao Municipal Hospital, Yao, Japan

**Keywords:** Atrial fibrillation, D-dimer, Direct oral anticoagulants, Left atrium

## Abstract

**Background:**

Atrial fibrillation (AF) may cause cerebral and systemic embolism. An increased D-dimer level indicates hyperactivation of secondary fibrinolysis, resulting in predilection for thrombosis. To clarify the differential effects of anticoagulation therapy, we compared the D-dimer levels in peripheral and left atrial (LA) blood of atrial fibrillation patients scheduled for ablation.

**Methods:**

We analyzed 141 patients with non-valvular AF (dabigatran, n = 30; apixaban, n = 47; edoxaban, n = 64; mean age: 68 years, male: 60%). Peripheral venous blood and LA blood was collected before pulmonary vein isolation. We examined the laboratory and echocardiographic parameters.

**Results:**

After adjusting for baseline characteristics, D-dimer level in the LA was significantly higher in patients treated with edoxaban than that in those on apixaban (0.77 ± 0.05 vs. 0.60 ± 0.05 μg/mL, *P* = 0.047), although there were no significant differences in peripheral D-dimer levels. We classified the D-dimer value of the LA into a normal group (< 0.9) and a high value group (≥ 1.0); the peripheral prothrombin fragment F1 + 2 level (odds ratio [OR] 1.012; 95% confidence interval [CI]: 1.003–1.022; *P* = 0.008) and left ventricular ejection fraction (LVEF) (OR, 0.947; 95% CI, 0.910–0.986; *P* = 0.008) were potential predictors of high LA D-dimer levels.

**Conclusions:**

In apixaban-treated patients, the D-dimer level in the left atrium was lower than in edoxaban-treated patients on the day of ablation, suggesting that the anticoagulant effect of apixaban on the left atrium is better than that of edoxaban in patients with AF.

## Introduction

Atrial fibrillation (AF) is a common arrhythmia associated with a prothrombotic or hypercoagulable state, which may increase the risk of cerebral and systemic embolism [1]. In AF patients, most of the thrombus is formed in the left atrium (LA).

It is well known that a hypercoagulative state is demonstrated by high levels of C-reactive protein (CRP) and D-dimer [2]. Increased D-dimer levels indicate hyperactivation of secondary fibrinolysis, meaning a tendency for intravascular thrombosis [3]. D-dimer is a product of cross-linked fibrin degradation, and it is a circulating marker of thrombogenesis and thrombus turnover [4]. Increasing D-dimer levels may reflect atrial thrombus formation and higher embolic risk in patients with AF. D-dimer is universally considered a gold standard test for activation of coagulation.

Catheter ablation is a well-established treatment option for patients with symptomatic AF [5]. Apart from vitamin K antagonists, the direct oral anticoagulants (DOACs) dabigatran, rivaroxaban, apixaban, and edoxaban have been approved for oral anticoagulation in patients with non-valvular AF [6]. Thus, there are different treatment possibilities for peri-interventional period anticoagulation in the setting of catheter ablation for AF.

Our aim was to assess the difference in coagulation status of the left atrium and related factors that impact patients with AF treated with different anticoagulants.

## Methods

This was a single-center, prospective, nonrandomized study aimed at identifying the antithrombin effect of the baseline steady state of anticoagulation treatment in patients with AF. We enrolled consecutive patients with AF aged > 20 years, who underwent ablation at the Yao Municipal Hospital between February 2018 and June 2019, followed 12 months after enrollment.

Exclusion criteria were a history of severe valvular heart disease, acute heart failure, thromboembolism, electrical defibrillation, trauma, and infection within 3 months. Patients receiving underdose and overdose of DOACs were also excluded. All patients underwent baseline transthoracic echocardiography (TTE) within 1 week and transesophageal echocardiography (TEE) before the ablation procedure. Baseline demographics and clinical information were obtained, and laboratory examinations were performed before catheter ablation. All patients underwent anticoagulation with dabigatran, apixaban, and edoxaban ≥ 4 weeks before the procedure (dabigatran 150 mg/110 mg b.i.d., apixaban 5 mg/2.5 mg b.i.d., edoxaban 60 mg/30 mg s.i.d.). Rivaroxaban is not included in this study because the standard dose of rivaroxaban (15 mg/10 mg s.i.d.) in Japan differs from the international standard dose (20 mg/15 mg s.i.d.). Anticoagulants were stopped only on the day of ablation. After ablation, all patients were followed up for 12 months to observe the presence or absence of thromboembolism.

Written informed consent was provided by all patients before participation, and the study protocol was approved by the institutional ethics committee.

Peripheral venous blood samples were collected immediately before the ablation procedure. For the clinical ablation procedure, a conventional single transseptal puncture was performed using an SL-1 sheath and a BRK-1 needle (St. Jude Medical Inc., Sunnyvale, CA, USA). Immediately after transseptal puncture and before heparin administration, a left atrial blood sample was simultaneously collected from the left atrial sheath by a cardiologist.

TTE was performed within a week before catheter ablation using a Philips Sonos 7500 ultrasound instrument (Philips Healthcare, Amsterdam, The Netherlands) equipped with a sector transducer (carrier frequency of 2.5 or 3.75 MHz). A 5-MHz phased-array multiplane probe was used for TEE. TTE parameters at baseline included left ventricular diastolic dimension (LVDd), ejection fraction (EF), left atrial diameter (LAD), and left atrial volume index (LAVI). TEE parameters at baseline included left atrial appendage (LAA) peak emptying velocity and presence of spontaneous echo contrast (SEC).

The following plasma/serum biomarkers were analyzed centrally at baseline: (1) thrombogenesis/fibrinolysis biomarkers: D-dimer, fibrinogen, prothrombin fragment F1 + 2, protein C, thrombomodulin, and (2) inflammation biomarker: CRP. For the quantitative measurement of D-dimer, a latex-enhanced photometric immunoassay (LPIA, Mitsubishi Chemical Medience Corporation, Tokyo, Japan) was used with an automatic analyzer (LPIA-S500). The detection limit of this assay was 0.3 μg/mL. The association of demographic data and medical history on biomarker levels at baseline was investigated. In addition, we assessed the relationship between anticoagulants, biomarker levels, and echocardiogram characteristics.

### Statistical Analysis

Data are expressed as mean and SD or median with 25th to 75th percentiles for normally distributed and skewed variables, respectively. Normality was assessed using the Shapiro–Wilk test. We used t-tests and chi-squared tests to compare continuous and categorical variables. To assess differences among the three groups, categorical variables were compared with the χ2 test, while continuous variables were compared using the Kruskal–Wallis test. Propensity score analysis was performed to determine the effect of the drug on D-dimer levels. The multiplicity of the tests was corrected by the Bonferroni method. A *P* value < 0.05 was considered significant. Statistical analysis was carried out using IBM SPSS (version 26.0, SPSS, Inc., Chicago, Illinois).

## Results

**Fig. 1:**
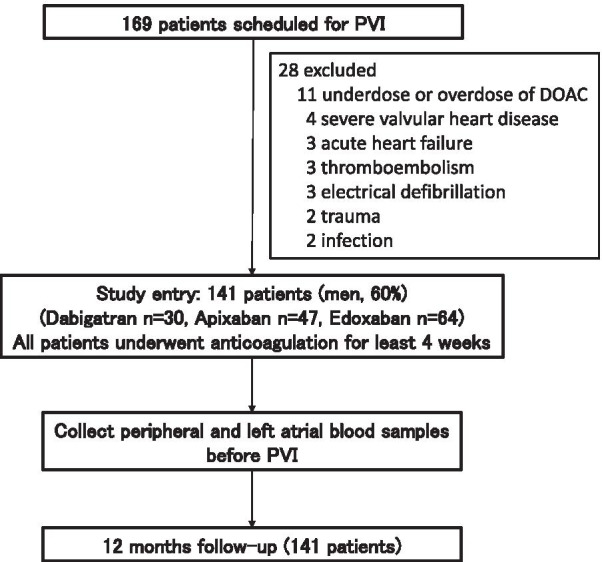
Study flow. PVI: pulmonary vein isolation

During entry periods, 169 patients with atrial fibrillation were initially screened, and finally 141 patients (on dabigatran n = 30, on apixaban n = 47, on edoxaban n = 64) were recruited (Fig. 1). The study population included 84 (60%) men, and the mean age at the baseline examination was 68.1 ± 10.1 years. Sixty-two (44%) patients had persistent AF. The mean CHADS2-VASc score was 2.4 ± 1.4. There were significant differences among the three groups in terms of gender, age, and history of heart failure. There was no significant difference in the frequency of use of angiotensin-converting enzyme inhibitors/angiotensin receptor blockers, β-blockers, statins, and anti-platelet drugs among the three groups (Table 1).

**Table 1:** Comparison of baseline characteristics among patients on dabigatran, apixaban, and edoxaban

	Total (n = 141)	Dabigatran (n = 30)	Apixaban (n = 47)	Edoxaban (n = 64)	*P* value
Male sex (%)	84 (60)	24 (80)	29 (62)	31 (48)	0.014
Age, years	68.1 ± 10.1	62.2 ± 9.0	70.4 ± 9.6	69.2 ± 10.0	0.001
Body weight, kg	65.0 ± 13.7	69.8 ± 15.8	64.8 ± 12.7	62.8 ± 13.0	0.070
BMI	24.6 ± 4.1	25.5 ± 4.9	24.7 ± 3.9	24.3 ± 4.0	0.459
AF type, persistent AF (%)	62 (44)	16 (53)	17 (36)	29 (45)	0.323
Congestive heart failure (%)	35 (25)	13 (43)	8 (17)	14 (22)	0.026
Hypertension (%)	80 (57)	16 (53)	30 (64)	34 (53)	0.488
Diabetes mellitus (%)	14 (10)	4 (13)	3 (6)	7 (11)	0.573
Stroke, TIA (%)	4 (3)	0 (0)	3 (6)	1 (2)	0.185
Vascular disease	5 (4)	1 (3)	2 (4)	2 (3)	0.949
Smoking	28 (20)	7 (23)	8 (17)	13 (20)	0.791
CHADS2 score	1.6 ± 1.0	1.6 ± 1.1	1.7 ± 1.0	1.6 ± 1.0	0.827
CHADS2-VASc score	2.4 ± 1.4	1.9 ± 1.2	2.6 ± 1.4	2.5 ± 1.4	0.063
ACEi/ARB (%)	46 (33)	7 (23)	17 (36)	22 (34)	0.466
β-blocker (%)	59 (42)	11 (37)	22 (47)	26 (41)	0.657
Statin (%)	30 (21)	5 (17)	12 (26)	13 (20)	0.632
Anti-platelet	8 (6)	1 (3)	4 (9)	3 (5)	0.584
Serum creatinine (mg/dL)	0.84 ± 0.23	0.84 ± 0.20	0.86 ± 0.25	0.82 ± 0.24	0.699
CrCl, mL/min	78.7 ± 34.4	94.8 ± 46.2	74.0 ± 28.2	74.5 ± 30.1	0.078
Hemoglobin, g/dL	13.9 ± 1.7	15.0 ± 1.3	13.5 ± 1.8	13.7 ± 1.7	0.001
BNP, pg/mL	154.5 ± 186.8	121.9 ± 137.7	163.4 ± 241.2	163.3 ± 160.3	0.562
Peripheral D-dimer, µg/mL	0.52 ± 0.29	0.41 ± 0.19	0.49 ± 0.27	0.60 ± 0.33	0.008
LA D-dimer, µg/mL	0.67 ± 0.35	0.51 ± 0.24	0.62 ± 0.39	0.78 ± 0.34	0.001
CRP systemic, mg/dL	0.17 ± 0.31	0.13 ± 0.17	0.20 ± 0.43	0.16 ± 0.25	0.583
Fibrinogen, mg/dL	270.5 ± 49.4	266.8 ± 48.1	267.5 ± 53.0	274.5 ± 47.8	0.690
Prothrombin fragment F1 + 2, pmol/L	125.0 ± 54.0	109.8 ± 23.1	127.4 ± 57.1	130.4 ± 61.0	0.021
Protein C	103.5 ± 26.1	130.1 ± 26.5	102.1 ± 24.3	91.9 ± 16.9	< 0.001
Thrombomodulin, ng/mL	2.4 ± 0.7	2.2 ± 0.6	2.6 ± 0.7	2.4 ± 0.8	0.122
LVDd, mm	46.1 ± 5.3	47.2 ± 6.2	45.9 ± 4.7	45.8 ± 5.2	0.420
LVEF, %	64.0 ± 10.5	64.0 ± 9.7	64.9 ± 10.1	63.3 ± 11.2	0.770
LAD, mm	42.0 ± 6.9	43.1 ± 7.9	41.2 ± 5.9	42.1 ± 7.0	0.479
LAVI, mL/m^2^	39.0 ± 14.6	37.0 ± 11.1	37.4 ± 12.1	41.2 ± 17.3	0.281
LAA flow velocity, cm/s	36.3 ± 19.4	40.1 ± 20.6	29.3 ± 11.8	39.5 ± 22.1	0.014
SEC( +), n (%)	35 (25)	10 (31)	16 (34)	10 (17)	0.119

Data are presented as mean ± SD

BMI, body mass index; AF, atrial fibrillation; TIA, transient ischemic attack; CHADS2 score, Congestive heart failure, Hypertension, Age ≥ 75, Diabetes mellitus, prior Stroke or transient ischemic attack (TIA); CHADS2-VASc score, Congestive heart failure, Hypertension, Age ≥ 75, Diabetes mellitus, prior Stroke or transient ischemic attack (TIA), vascular disease, age (65–74), sex category; ACEi, angiotensin converting enzyme inhibitor; ARB, angiotensin-receptor blocker; CrCl, creatinine clearance; BNP, brain natriuretic peptide; CRP, C-reactive protein; LA, left atrial; LVDd, left ventricular end-diastolic dimension; LVEF, left ventricular ejection fraction; LAD, left atrial dimension; LAVI, left atrial volume index; LAA, left atrial appendage; SEC, spontaneous echo contrast

Laboratory and echocardiographic data of all patients are shown in Table 1. Serum creatinine and CRP levels, and brain natriuretic peptide levels in peripheral blood were not significantly different between the three groups. Echocardiographic parameters (LVDd, EF, LAD, LAVI, and SEC) did not differ among them. There were significant differences among the three groups in hemoglobin concentration and LAA flow velocity (Table 2).

**Table 2:** Adjusted average and 95% CI according to medications

	① Dabigatran (n = 30)		② Apixaban (n = 47)		③ Edoxaban (n = 64)		*P* value		
	Average	95% CI	Average	95% CI	Average	95% CI	① vs ②	① vs ③	② vs ③
Peripheral D-dimer, μg/mL	0.523	0.397–0.648	0.480	0.396–0.564	0.551	0.476–0.626	1.000	1.000	0.633
LA D-dimer, μg/mL	0.550	0.397–0.704	0.603	0.501–0.706	0.774	0.682–0.866	1.000	0.074§	0.047*

^*^: *P* < 0.05, §: *P* < 0.1

CI, confidence interval. Other abbreviations as in Table 1

Fibrinogen and thrombomodulin levels were not significantly different among the three groups. However, there were significant differences in the D-dimer levels of the left atrium (*P* = 0.001). The prothrombin fragment 1 + 2 level of peripheral blood in the dabigatran group was lower among the three groups (*P* = 0.021). The protein C level of peripheral blood in the edoxaban group was markedly lower (*P* < 0.001).

**Fig. 2:**
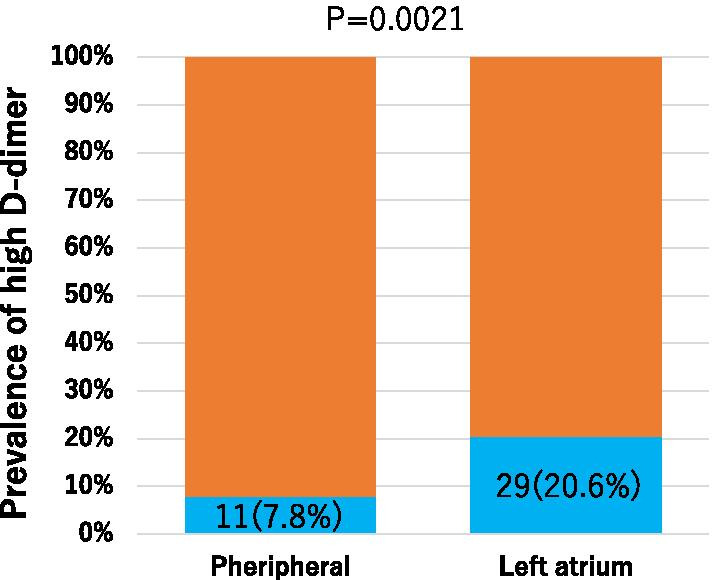
Prevalence of high D-dimer levels in the peripheral circulation and the left atrium. The prevalence of high D-dimer levels (≥ 1.0 µg/mL) was significantly greater in the left atrium than in the peripheral circulation

**Fig. 3:**
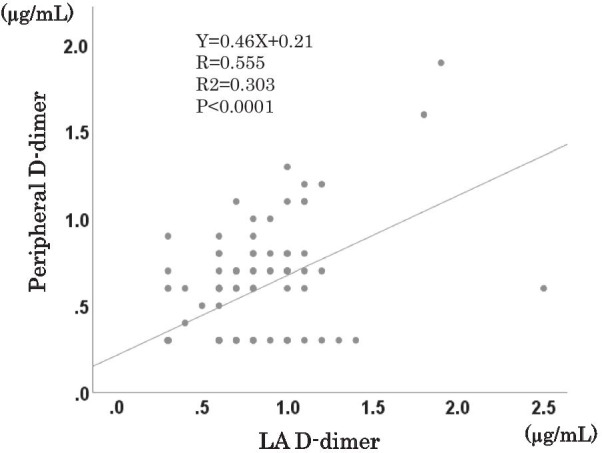
Association between peripheral and left atrial (LA) D-dimer levels. A moderate correlation was found between peripheral and LA D-dimer levels

In this study, the prevalence of high D-dimer levels (≥ 1.0 µg/mL) was significantly higher in the LA than in the periphery (20.6% vs. 7.8%, *P* = 0.002) (Fig. 2). We performed a single regression analysis of systemic and LA D-dimer, and found a moderate correlation between the two parameters (r = 0.560, *P* < 0.001) (Fig. 3).

The effects of medication with dabigatran, apixaban, or edoxaban on peripheral D-dimer and LA D-dimer were analyzed by propensity score analysis. Table 2 shows the standardized function coefficients of the canonical discriminant analysis performed to calculate the propensity score. There were no differences among the three groups with respect to peripheral D-dimer levels. The D-dimer level in the LA was significantly lower in the apixaban group than in the edoxaban group (*P* = 0.047). On the other hand, the D-dimer level in the LA tended to be lower in the dabigatran group than in the edoxaban group (*P* = 0.074).

We classified the patients into two groups according to the D-dimer values of the LA i.e., less than 1.0 and 1.0 or more, and compared their characteristics (Table 3). No significant differences were observed between the percentage of persistent AF, CHADS2-VASc score, hemoglobin concentration, and renal function. Fibrinogen levels were significantly higher in the high D-dimer group than in the normal D-dimer group (287.1 ± 56.4 vs. 266.2 ± 46.8, *P* = 0.043). Prothrombin fragment F1 + 2 levels were significantly higher in the high D-dimer group than in the normal D-dimer group (157.0 ± 84.7 vs. 116.6 ± 39.0, *P* = 0.018).

**Table 3:** Comparison of normal and high of the D-dimer levels in the LA

	Normal LA D-dimer < 0.9 µg/mL (n = 112)	High LA D-dimer ≥ 1.0 µg/mL (n = 29)	*P* value
Age, year	67.6 ± 10.1	70.0 ± 10.2	0.262
Body weight, kg	64.8 ± 13.4	65.8 ± 15.0	0.734
BMI	24.6 ± 4.1	24.9 ± 4.3	0.731
Persistent AF (%)	49 (44)	13 (45)	0.917
CHADS2 score	1.63 ± 1.04	1.62 ± 0.90	0.984
CHADS2-VASc score	2.38 ± 1.36	2.55 ± 1.48	0.546
Hemoglobin, g/dL	14.0 ± 1.7	13.5 ± 2.0	0.158
Serum creatinine (mg/dL)	0.83 ± 0.23	0.86 ± 0.25	0.372
CLCr, mL/min	79.1 ± 33.1	76.8 ± 39.5	0.748
BNP, pg/mL	147.7 ± 174.3	180.9 ± 230.4	0.473
Systemic CRP, mg/dL	0.13 ± 0.19	0.31 ± 0.55	0.091
Fibrinogen, mg/dL	266.2 ± 46.8	287.1 ± 56.4	0.043
Prothrombin fragment F1 + 2, pmol/L	116.6 ± 39.0	157.0 ± 84.7	0.018
Protein C, %	105.4 ± 25.9	96.0 ± 26.1	0.084
Thrombomodulin, ng/mL	2.39 ± 0.60	2.62 ± 0.95	0.213
LVDd, mm	45.8 ± 5.1	47.3 ± 5.7	0.170
EF, %	65.2 ± 9.1	59.3 ± 13.7	0.037
LAD, mm	41.6 ± 6.7	43.7 ± 7.1	0.141
LAVI, mL/m^2^	38.3 ± 15.1	41.9 ± 12.4	0.249
LAA flow velocity, cm/s	0.37 ± 0.20	0.35 ± 0.16	0.618
Dabigatran, n (%)/Apixaban, n (%)/Edoxaban, n (%)	27(90)/40(85)/45(70)	3(10)/7(15)/19(30)	0.045

Data are presented as mean ± SD

Echocardiographic parameters, including LVDd, EF, LAD, LAVI, and LAA flow velocity, were not significantly different between the two groups. Left ventricular ejection fraction (LVEF) was significantly lower in the high D-dimer group than in the normal D-dimer group (59.3 ± 13.7 vs. 65.2 ± 9.1, *P* = 0.037). The proportion of patients treated with edoxaban was higher in the high D-dimer group than in the normal group (*P* = 0.045). The joint contribution of predictors of high LA D-dimer levels was assessed by multivariate analysis (Table 4). Prothrombin fragment F1 + F2 (odds ratio [OR] 1.012, 95% confidence interval [CI] 1.003–1.022, *P* = 0.008) and LVEF (OR 0.947, 95% CI 0.910–0.986, *P* = 0.008) were independent predictors of high LA D-dimer levels.

**Table 4:** Multivariate analysis of potential predictors of high LA D-dimer level

	OR	95% CI	*P* value
Fibrinogen, mg/dL	1.009	1.000–1.018	0.054
Prothrombin fragment F1 + 2, pmol/L	1.012	1.003–1.022	0.008
LVEF, %	0.947	0.910–0.986	0.008

OR, odds ratio. Other abbreviations as in Tables 1 and 2

All patients were followed up for 12 months after AF ablation. One patient in the edoxaban group had a transient ischemic attack (TIA) 9 months after ablation. A new cerebral infarction was detected on head computed tomography, but fortunately, no sequelae remained.

## Discussion

Three new facts emerged from this study. (1) Systemic D-dimer level had a good correlation with LA D-dimer level, and it was lower than the LA D-dimer level in patients treated with DOACs. (2) There were no differences in peripheral D-dimer levels among patients with dabigatran, apixaban, and edoxaban. However, the LA D-dimer level was significantly lower in the apixaban group than in the edoxaban group. (3) Peripheral prothrombin fragment F1 + F2 levels and LVEF were potential predictors of high LA D-dimer levels.

It has been reported that elevated D-dimer levels are associated with thromboembolism [2, 7], but a difference in D-dimer level was found in blood collected from the LA where thrombus formation readily occurs. Another clinical study demonstrated that LA D-dimer levels were higher than systemic D-dimer levels in patients with paroxysmal AF receiving warfarin [8]. Our study found that D-dimer levels in the left atrium were higher than those in the peripheral circulation in patients treated with DOAC.

Major phase III clinical trials for prevention of stroke or systemic embolic events in patients with AF showed that dabigatran and apixaban significantly reduced the risk of stroke and systemic embolic events compared to warfarin, but edoxaban failed to show superiority [6, 9, 10]. Thereafter, several meta-analyses have reported that dabigatran and apixaban were superior to warfarin in prevention of stroke and systemic embolism [11]. Another meta-analysis reported that apixaban was overall superior to other DOACs in the prevention of stroke, systemic embolism, and caused fewer bleeding complications [12].

We previously reported that a direct thrombin inhibitor, dabigatran, administered twice a day was effective in resolving preexisting thrombi and would be more effective in reducing thrombus formation than a factor Xa inhibitor administered once a day [13].

In this study, peripheral prothrombin fragment F1 + 2 levels were higher in the high LA D-dimer group than in the normal D-dimer group. Prothrombin fragment F1 + 2 is a peptide released from prothrombin upon conversion from prothrombin to thrombin, reflecting 100% thrombin generation. It accurately reflects the amount of thrombin produced [14, 15]. This considered to be a state in which thrombus is more likely to occur in the high D-dimer group than in the normal D-dimer group. In this study, low LVEF was a predictor of high LA D-dimer levels. A previous study reported that decreased LVEF led to LA thrombus formation [16, 17].

In atrial failure through atrial cardiomyopathy, thromboembolic risk seems to be associated with fibrosis degree [18]. Previously reported that late-gadolinium enhancement cardiac magnetic resonance imaging (LGE-MRI) examination of structural remodeling of the left atrium, structural remodeling of the left atrium was more common in the group with reduced LVEF [19]. AF patients with a high degree of LA structural remodeling may have low baseline LVEF and have LA fibrosis [20].

The LA D-dimer value was significantly higher in the edoxaban group, and conversely, the proportion of edoxaban-treated patients was higher in the high LA D-dimer group. According to a meta-analysis comparing phase III study evidence, the twice-daily dosing regimen of non-vitamin K antagonist oral anticoagulants appears to offer a more balanced risk–benefit profile with respect to stroke prevention and intracranial hemorrhage [21]. The half-life of dabigatran is 12 to 17 h, of apixaban is 9 to 14 h, and of edoxaban is 9 to 11 h [22]. Of the three drugs, edoxaban has the shortest half-life, but is used at a once-daily dosing regimen. It is possible that a decrease in the blood concentration of the drug several hours after administration effected the increase in LA D-dimer levels.

DOACs have been shown to prevent thromboembolism in patients with non-valvular AF. Compared to warfarin, DOACs have the advantage that they can be used without regular coagulation monitoring and dose adjustment, but they also have the disadvantage that coagulation monitoring cannot be performed easily, as with warfarin [23]. In the present study, the incidence of thromboembolism was only 1 case during the 12-month follow-up period after ablation. During the 12-month observation period after ablation, 121 of 141 (86%) patients maintain sinus rhythm. The large number of patients who maintained sinus rhythm may have contributed to the low incidence of thromboembolism during the 12-month follow-up period. However, in patients with a high risk of stroke and systemic embolism, regular D-dimer, prothrombin fragment F1 + 2, and LVEF check should be performed to determine the presence or absence of hypercoagulability [19–22].

### Study limitations

First, the number of patients in this study was small. Second, the choice of anticoagulant depended on the judgment of each doctor and was not randomized. Third, we did not measure anti-Xa activity. The reason for not measuring anti-Xa activity is that dabigatran does not inhibit factor Xa. Another reason is that it has been previously reported that the anti-Xa activity measured in anti-Xa drugs does not completely reflect the overall biological spectrum of anti-Xa drugs [24]. Fourth, the incidence of asymptomatic thromboembolism was unknown because we did not perform whole-body CT or MRI on asymptomatic patients. It is essential to identify AF patients whose D-dimer levels of left atrium are high. Fifth, we did not perform LGE-MRI. Therefore, the degree of fibrosis and structural remodeling of the left atrium involved in thromboembolism have not been evaluated. A multicenter randomized trial is necessary to confirm the accuracy of the present results and the incidence of thromboembolic events.

## Conclusions

Measurements of D-dimer, prothrombin fragment F1 + 2, and LVEF may be effective in confirming hypercoagulability in patients with AF taking DOACs. In the apixaban group, LA D-dimer level was lower than in the edoxaban group, suggesting that the anticoagulant effect of apixaban is better than that of edoxaban in patients with non-valvular AF.

## Data Availability

The datasets used or analyzed during the current study are available from the corresponding author on reasonable request.

## Abbreviations

AF: Atrial fibrillation
CRP: C-reactive protein
DOAC: Direct oral anticoagulant
EF: Ejection fraction
LA: Left atrial
LAA: Left atrial appendage
LAD: Left atrial diameter
LAVI: Left atrial volume index
LPIAL: Latex-enhanced photometric immunoassay
LVDd: Left ventricular diastolic dimension
MRI: Magnetic resonance imaging
PVI: Pulmonary vein isolation
SEC: Spontaneous echo contrast
TEE: Transesophageal echocardiography
TTE: Transthoracic echocardiography
